# Three-dimensional electron microscopy for endothelial glycocalyx observation using Alcian blue with silver enhancement

**DOI:** 10.1007/s00795-020-00267-1

**Published:** 2020-10-06

**Authors:** Shumpei Mukai, Takashi Takaki, Tasuku Nagumo, Mariko Sano, Dedong Kang, Masafumi Takimoto, Kazuho Honda

**Affiliations:** 1 Department of Pathology, Showa University School of Medicine, Tokyo, Japan; 2 Department of Anatomy, Showa University School of Medicine, 1-5-8 Hatanodai, Shinagawa-ku, Tokyo, 142-8555 Japan; 3 Division of Electron Microscopy, Showa University, Tokyo, Japan

**Keywords:** Glycocalyx, Alcian blue, Silver enhancement, PAM stain, Low-vacuum scanning electron microscopy, Formalin-fixed paraffin embedding, Correlative light and electron microscopy

## Abstract

Glycocalyx (GCX) is a thin layer of negatively charged glycoproteins that covers the vascular endothelial surface and regulates various biological processes. Because of the delicate and fragile properties of this structure, it is difficult to detect GCX morphologically. We established a simple method for a three-dimensional visualization of endothelial GCX using low-vacuum scanning electron microscopy (LVSEM) on formalin-fixed paraffin-embedded (FFPE) sections. Mouse kidney tissue was fixed with 10% buffered formalin containing 1% Alcian blue (ALB) via perfusion and immersion. FFPE sections were observed by light microscopy (LM) and LVSEM, and formalin-fixed epoxy resin-embedded ultrathin sections were observed by transmission electron microscopy (TEM). The endothelial GCX from various levels of kidney blood vessels was stained blue in LM and confirmed as a thin osmiophilic layer in TEM. In LVSEM, the sections stained by periodic acid methenamine silver (PAM) revealed the endothelial GCX as a layer of dense silver-enhanced particles, in both the samples fixed via perfusion and immersion. Correlative light and electron microscopy (CLEM) revealed the fine visible structure of endothelial GCX. This simple method using FFPE samples with ALB will enable the three-dimensional evaluation of endothelial GCX alterations in various human diseases associated with endothelial injury in future studies.

## Introduction

Endothelial glycocalyx (GCX) is a thin layer of glycoproteins covering the luminal surface of vascular endothelial cells and is associated with various important biological functions, including cell adhesion, migration, differentiation, and inflammation [1–4]. Because of its fragile properties, visualization of endothelial GCX is difficult using traditional light and electron microscopic procedures. To combat this issue, several techniques using cationic molecules that bind to negatively charged GCX, such as lanthanum [5–9], Alcian blue (ALB) [5, 6, 8, 10–12], and ruthenium red [12, 13], have been developed previously for use with transmission electron microscopy (TEM). Recently, a method using scanning electron microscopy (SEM) has also been reported [9]. Moreover, several novel methods have been developed for the use of frozen non-fixed ultrathin sections for TEM [14, 15] and to observe lectins by immunofluorescent microscopy [16–21]. Electron microscopy (EM) is a useful tool that allows visualization of structures at a higher resolution. However, the procedures to prepare samples for TEM, especially for embedding and sectioning, are time and labor intensive and require special laboratory facilities and technical skills. Therefore, we developed a simple method to visualize endothelial GCX in three dimensions using low-vacuum SEM (LVSEM) of formalin-fixed paraffin-embedded (FFPE) sections. The optimal conditions for fixation with ALB were investigated to detect endothelial GCX using LVSEM, light microscopy (LM), and correlative light and electron microscopy (CLEM). These methods allow investigation of alterations in the endothelial glycocalyx in various human diseases associated with endothelial injury.

## Materials and methods

### Animals

Fifteen 10-week-old female BALB/c mice (Sankyo Labo Service, Tokyo, Japan) were used in this study. Mice were divided into three groups: five for ALB perfusion fixation, five for 10% neutral buffered formalin perfusion fixation, and five for ALB and 10% neutral buffered formalin immersion fixation. This study was performed according to the regulations of the animal experiment committee of the Showa University (Animal experiment approval number # 09100).

### Fixation

#### Fixative

A solution consisting of 10% neutral buffered formalin (FUJIFILM Wako Pure Chemical, Osaka, Japan), 1% ALB 8GX (Sigma-Aldrich Japan, Tokyo, Japan), and 2% sucrose was used as ALB fixative. As the solubility and staining properties of ALB change depending on the pH of the solvent, ALB-containing fixatives were adjusted from pH 1 to 7 by adding a 100% acetic acid solution. The osmolality of ALB fixative and ALB ( −) fixative was measured using osmotic pressure analyzer, OSMO STATION (OM-6060, ARKSRAY, Tokyo, Japan), revealing an osmolality of 1751 and 1670 mOsm/L in ALB fixative and ALB ( −) fixative, respectively. Specimens were immersed in ALB fixative at pH 1.0, 3.0, 4.0, 5.0, 5.5, and 6.0 for 5 days at 4 °C to determine the optimal pH, and the optimal pH was determined as pH 6.0 (see Results and Fig. 2). Perfusion fixation was also performed using ALB fixative at pH 6.0.

#### Perfusion fixation

**Fig. 1:**
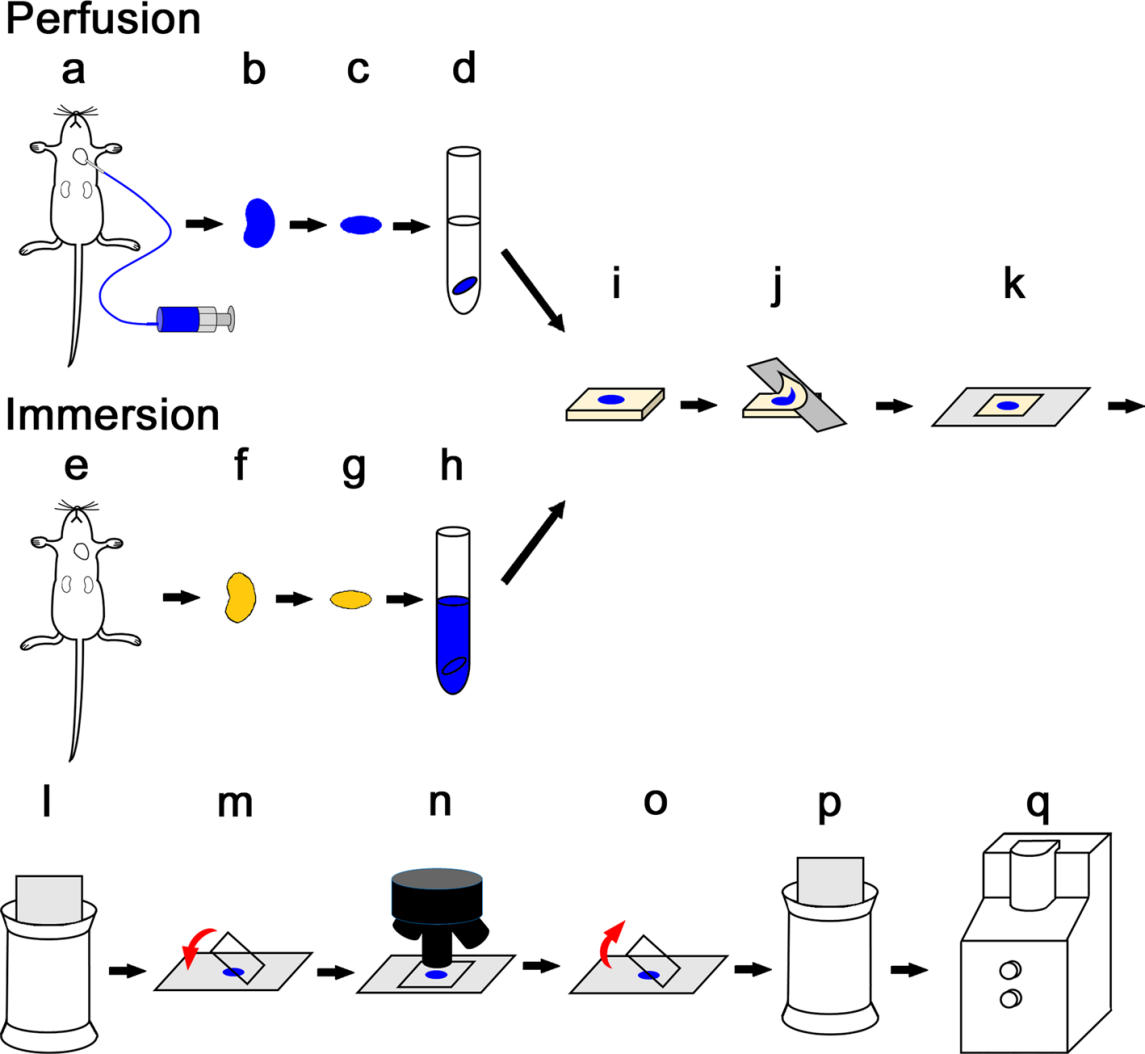
Sample preparation procedures and CLEM. Mice were perfused with 10% neutral buffer formalin, 2% sucrose, and 1% Alcian blue (ALB fixative, pH 6) (**a**) or euthanized without perfusion (**e**). The kidneys were excised (**b**, **f**), cut into 1 mm thickness (**c**, **g**), and immersed in 10% neutral buffer formalin with 2% sucrose overnight (**d**) or ALB fixative for 5 days at 4 °C (**h**). The kidneys were embedded in paraffin (**i**) and sliced at 2 μm thickness (**j**). After deparaffinization (**l**), they were covered with cover glasses (**m**) and scanned using a virtual slide system (**n**). After removing the cover glasses (**o**), they were stained with PAM stain (**p**) and observed using low-vacuum scanning electron microscopy (LVSEM) (**q**)

Mice were anesthetized by intraperitoneal injection of medetomidine (0.3 mg/kg, Meiji Seika Pharma, Tokyo, Japan), midazolam (4 mg/kg, SANDOZ, Tokyo, Japan), and butorphanol tartrate (2.5 mg/kg, Meiji Seika Pharma, Tokyo, Japan) combination. After pre-perfusion with 0.1 M phosphate buffer solution containing 2% sucrose for 3 min, the anesthetized mice were perfused with ALB fixative (pH 6.0) through a cannula placed in the left ventricle at 2 ml/min for 10 min, using a perfusion pump (Fig. 1a). The right atrium was incised for drainage of the perfused solution. The kidneys were then excised (Fig. 1b), cut to 1 mm thickness (Fig. 1c) and immersed in 10% neutral buffered formalin fixative at 4 °C overnight (Fig. 1d).

#### Immersion fixation

Mice were anesthetized as described previously and euthanized via blood removal through a right atrial incision (Fig. 1e). The kidneys were excised from the mice (Fig. 1f), cut into 1 mm thickness (Fig. 1g), and immersed in ALB fixative (pH 6.0) at 4 °C (Fig. 1h). Samples were immersed for 1, 3, 5, and 7 days to determine the optimal immersion time. Immersion fixation performed at room temperature failed to stain the samples because of precipitation of ALB.

### Capturing light microscopic images using the virtual slide system

Paraffin-embedded blocks were prepared for LM by the usual preparation methods (Fig. 1i) and were cut at 2 μm (Fig. 1j). After deparaffinization (Fig. 1l), samples were immersed in water, cover glassed (Fig. 1m), and scanned using the virtual slide system (VSS) (VS-100, OLYMPUS, Tokyo, Japan) (Fig. 1n).

### Periodic acid methenamine silver (PAM) stain enhancement for ALB

After removing the cover glass (Fig. 1o), slides were oxidized in 1% periodic acid solution for 10 min, incubated in Gomori’s methenamine silver solution for 70 min at 65 °C, incubated in gold chloride solution for 5 min, and treated with sodium thiosulfate solution for 2 min (Fig. 1p).

### Observation of endothelial GCX by LVSEM

After PAM staining, the specimen was air-dried and observed without metal coating by LVSEM (SU-1000; Hitachi High-Tech Co. Tokyo, Japan) using backscattered electron mode with acceleration voltage of 10 kV and a spot size 40 in 30 Pa (Fig. 1q).

### Correlative light and electron microscopy (CLEM)

The same area of the same sample was observed with VSS and LVSEM for CLEM. Each image was merged using image overlay verification software, AZblend (Astron, Tokyo, Japan).

### Observation of endothelial GCX by TEM

After perfusion fixation, kidneys were cut to 1 mm thickness and immersed in 10% neutral buffered formalin at 4 °C overnight. Kidneys were cut to 1 mm thickness and immersed in ALB fixative (pH 6.0) at 4 °C for 5 days. The specimens were washed in 0.1 M phosphate buffer solution and post-fixed with 1% osmium tetroxide at 4 °C for 2 h. The specimens were dehydrated through a graded ethanol series and embedded in epoxy resin. After ultrathin sectioning (70 nm), the sections were stained with 2% uranyl acetate and 2.7% lead citrate (Reynolds) and observed by TEM (H-7600; Hitachi High-Tech Co. Tokyo, Japan) at 80 kV of acceleration voltage.

### Measurement of endothelial GCX thickness by TEM

The GCX of the glomerular capillary (GC), peritubular capillary (PTC), small artery (SA), and small vein (SV) were imaged using TEM. The thickness of GCX was measured at 30 randomly selected points in the area using ImageJ (NIH). Measurement points were selected according to the orthogonally cut sections, which were confirmed by the shapes of epithelial foot processes and endothelial fenestrations. Tangentially cut sections were eliminated. The average GCX thickness was calculated, and the values are listed as the mean ± SD. JMP Pro (version 14.0.0, SAS Institute Inc, Cary, NC; 2018) was used for statistical analysis. Student’s t-tests were used to calculate statistical significance, and a p-value of less than 0.05 was considered statistically significant.

## Results

### Optimal conditions for ALB fixation

#### pH of ALB fixative (Fig. 2)

**Fig. 2:**
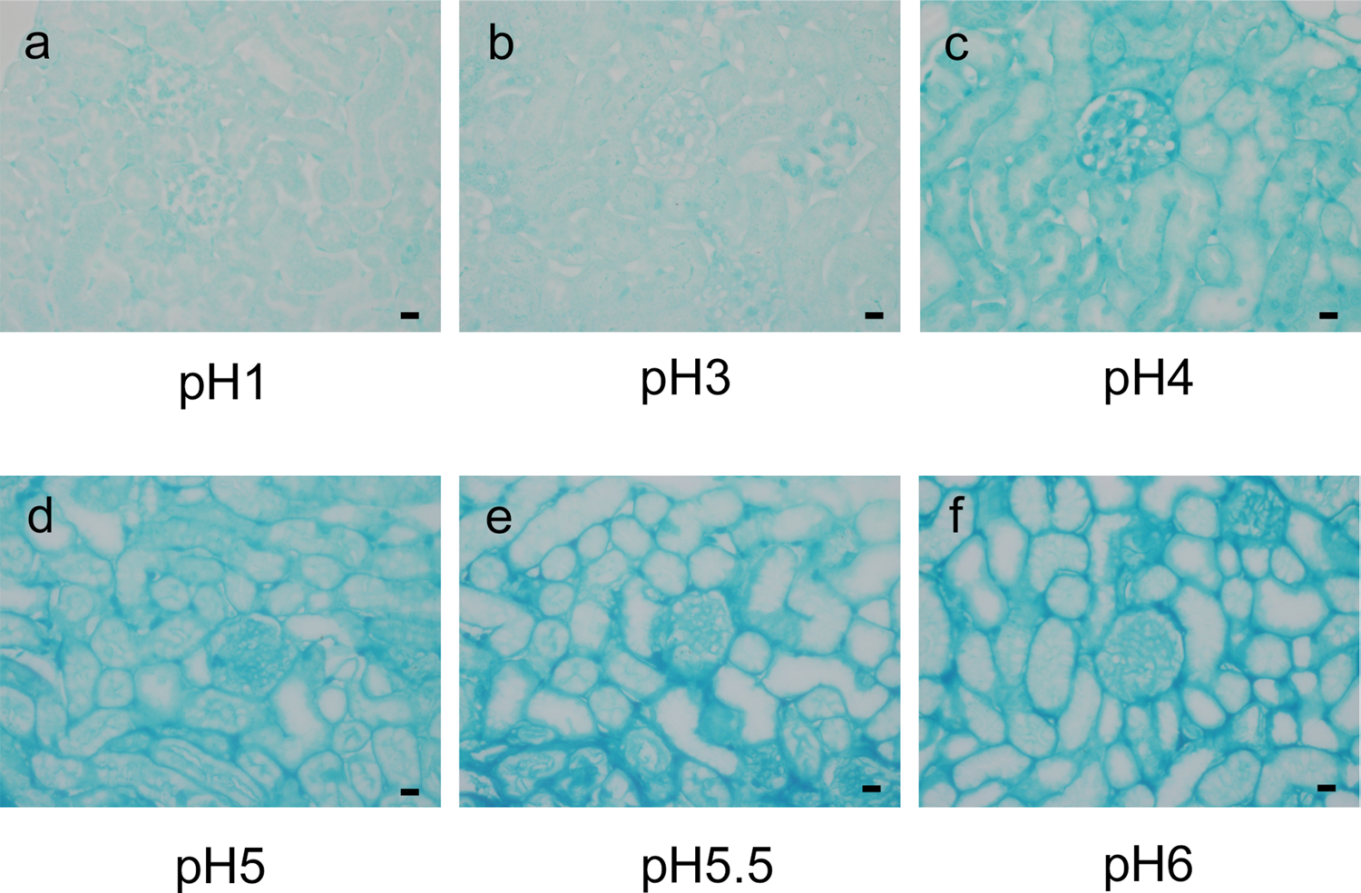
ALB staining of FFPE sections fixed in fixatives of different pH. The specimens were immersed in fixative solutions composed of 10% neutral buffer formalin, 2% sucrose, and 1% Alcian blue at different pH levels, including **a**: pH 1.0, **b**: pH 3.0, **c**: pH 4.0, **d**: pH 5.0, **e**: pH 5.5, and **f**: pH 6.0) for 5 days at 4 °C The specimens were poorly stained at pH levels 1.0 and pH 3.0; insufficiently stained at pH levels 4.0 and pH 5.0; and well-stained at pH levels 5.5 and pH 6.0. The optimum pH was determined to be 6.0, as it closely matches physiological pH. Magnification: 600 × . Scale bars: 10 μm

The specimens were poorly stained in ALB fixative at pH levels of 1.0 and 3.0 (Fig. 2a, b); insufficiently stained at pH levels of 4.0 and pH 5.0 (Fig. 2c, d); and well-stained at pH levels of 5.5 and 6.0 (Fig. 2e, f). Staining at pH 7.0 failed because of ALB precipitation. We determined that the optimal pH level was 6.0 because its neutrality more closely approximates that of blood. Perfusion fixation was also performed using ALB fixative at pH 6.0.

#### The optimal immersion time for ALB fixative (Fig. 3)

**Fig. 3:**
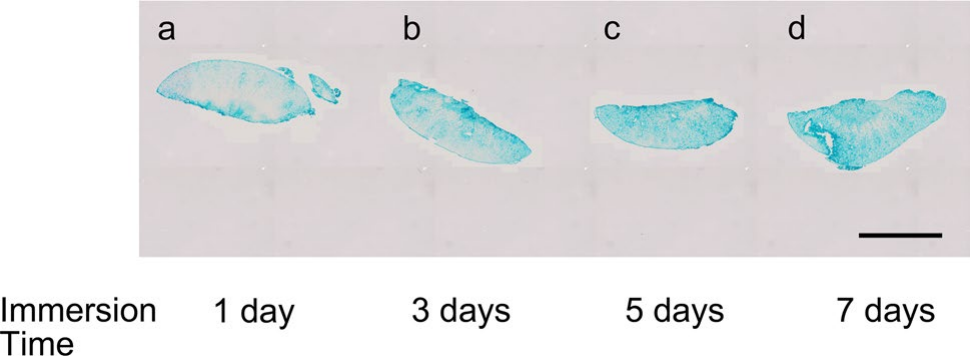
ALB staining of FFPE sections fixed in different times (1–7 days). The specimens were immersed in a solution composed of 10% neutral buffer formalin, 2% sucrose, and 1% Alcian blue (pH 6) for different durations, including **a**: 1 day, **b**: 3 days, **c**: 5 days, and **e**: 7 days. The sections stained non-homogeneously with immersion for 1 and 3 days and more homogenously with immersion for 5 and 7 days, without significant differences. The optical duration for immersion fixation was 5 days (magnification = 40 ×). Scale bars: 2 mm

The sections were insufficiently stained after immersion for 1 and 3 days in ALB fixative (Fig. 3a, b) and were almost homogenously stained after 5 and 7 days of immersion, without significant difference in staining features (Fig. 3c, d). Thus, 5 days was determined to be the optimal immersion period because it was a minimally required period for ALB permeation.

### Endothelial GCX imaging of GC by LVSEM using ALB perfusion and silver enhancement by PAM staining (Fig. 4)

**Fig. 4:**
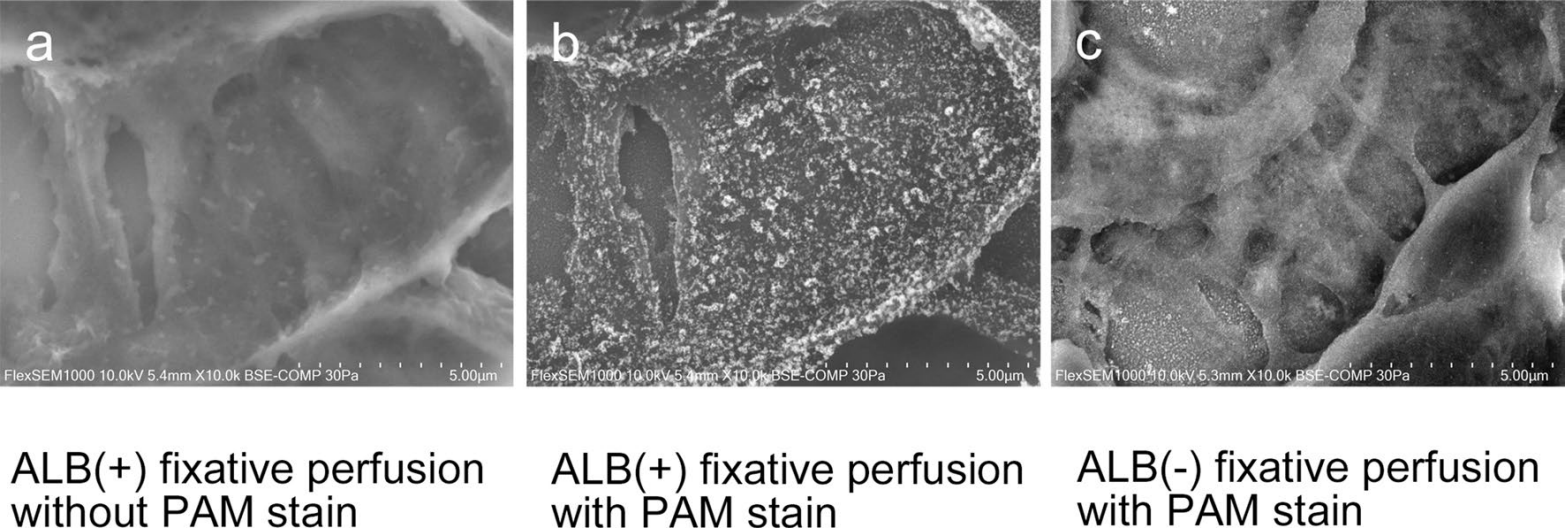
Endothelial GCX imaging of glomerular capillary by LVSEM using ALB perfusion with silver enhancement by PAM stain. LVSEM images of glomerular capillary (GC) perfused with ALB ( +) fixative without PAM stain (**a**), perfused with ALB ( +) fixative with PAM stain (**b**), and perfused with ALB ( −) fixative with PAM stain (**c**). Silver-enhanced particles that adhered to glomerular endothelium were visualized only in GC perfused with ALB ( +) fixative and subsequently stained with PAM (**b**). No adhesion of silver-enhanced particles was observed in GC perfused with ALB ( +) fixative without PAM stain (**a**) and in GC perfused with ALB ( −) fixative with PAM stain (**c**). The glomerular basement membrane (GBM) is the thin membranous structure seen in specimens stained with PAM (**c**); however, samples stained with PAM are obscure (**a**). Magnification **a**–**c**: 10,000 ×

LVSEM observation of GC perfused with ALB fixative revealed no particles on the surface of the capillary lumen when the section was not stained with PAM (Fig. 4a). However, fine granular particles, with diameters from 50 to 100 nm, were densely attached to the luminal surface of GC when the section was stained with PAM (Fig. 4b). No particles were observed in GC perfused with ALB ( −) fixative, even when the section was stained with PAM (Fig. 4c). The glomerular basement membrane (GBM) was faintly visible as a thin membranous structure in specimens stained with PAM (Fig. 4c), whereas specimens not stained with PAM appeared obscure (Fig. 4a). The fenestrations in glomerular epithelial cells were not visible via LVSEM because the cell membrane and the cellular cytoplasm were not stained with PAM. BSE signals from GBM and GCX can be distinguished from each other by differences in appearance. GBM was seen as a thin, membranous structure with fine BSE signals, whereas GCX appeared as particles with coarse and bright BSE signals (Fig. 4b).

### Endothelial GCX imaging of kidney vasculature perfused with ALB fixative

#### LM (Fig. 5)

**Fig. 5:**
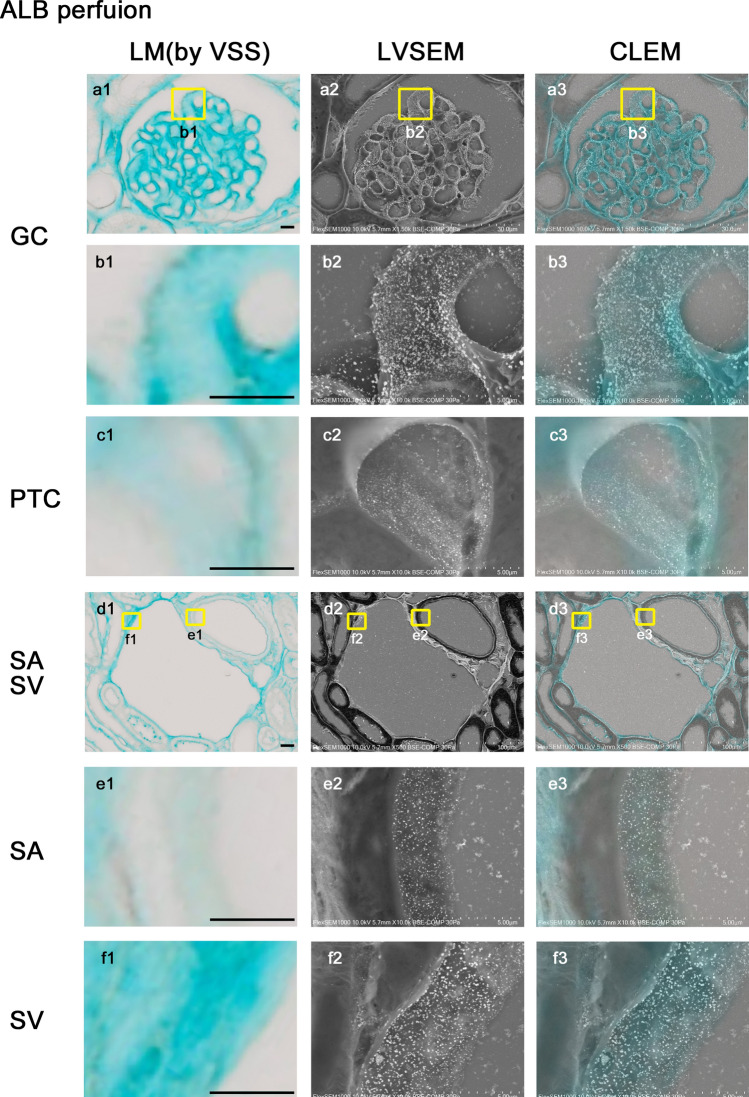
Endothelial GCX imaging of kidney vasculature perfused with ALB fixative and stained with PAM. The kidneys were perfused with ALB fixative composed of 10% neutral buffer formalin, 2% sucrose, and 1% ALB (pH 6) and were observed by light microscopy using a virtual slide system (**a1**–**f1**), LVSEM (**a2**–**f2**), and merged CLEM images (**a3**–**f3**). The vessel walls of the glomerular capillary (GC) (**a1**, **b1**), peritubular capillary (PTC) (**c1**), small artery (SA) (**d1**, **e1**), and vein (SV) (**d1**, **f1**) were stained in blue. The same position of GC (**a2**, **b2**), PTC (**c2**), SA (**d2**, **e2**), and SV (**d2**, **f2**) in the same section were observed by LVSEM after PAM stain. Silver particles that adhered to the vascular endothelial surfaces were observed clearly. LM and LVSEM images were merged into CLEM images, GC (**b3**), PTC (**c3**), SA (**e3**), and SV (**f3**). The positive site of ALB staining coincided with the site of silver-enhanced particle attachment. Magnifications: **a1**-3: 1500 × ; **b1**-3, **c1**-3: 10,000 × ; **d1**-3: 500 × ; **e1**-3, **f1**-3: 10,000 × . Scale bars: 5 μm

By perfusion of ALB fixative for 10 min, the glomerulus, Bowman’s capsule, and interstitium were stained in blue (Fig. 5a1). The inner surfaces of SA and SV were also stained in blue (Fig. 5d1). The detailed localization of ALB in GC (Fig. 5b1) and PTC (Fig. 5c1) was difficult to define because of the limit of the light microscopic resolution. The inner surface of SA (Fig. 5e1) was stained less than that of SV (Fig. 5f1).

#### LVSEM (Fig. 5)

In LVSEM, fine granular particles enhanced by silver were observed on the endothelial surfaces of GC (Fig. 5a2, b2) and PTC (Fig. 5c2). These particles were also found on the luminal surfaces of SA and SV (Fig. 5d2). The number of particles adhering to SA (Fig. 5e2) was less than that of SV (Fig. 5f2).

#### CLEM (Fig. 5)

Upon merging the light microscope and LVSEM images, the areas stained by ALB corresponded to the sites where the silver-enhanced particles were visualized by LVSEM (Fig. 5a3–f3). The CLEM images clearly demonstrated the site and state of endothelial GCX three-dimensionally in various levels of the kidney vasculature.

#### TEM (Fig. 6)

**Fig. 6:**
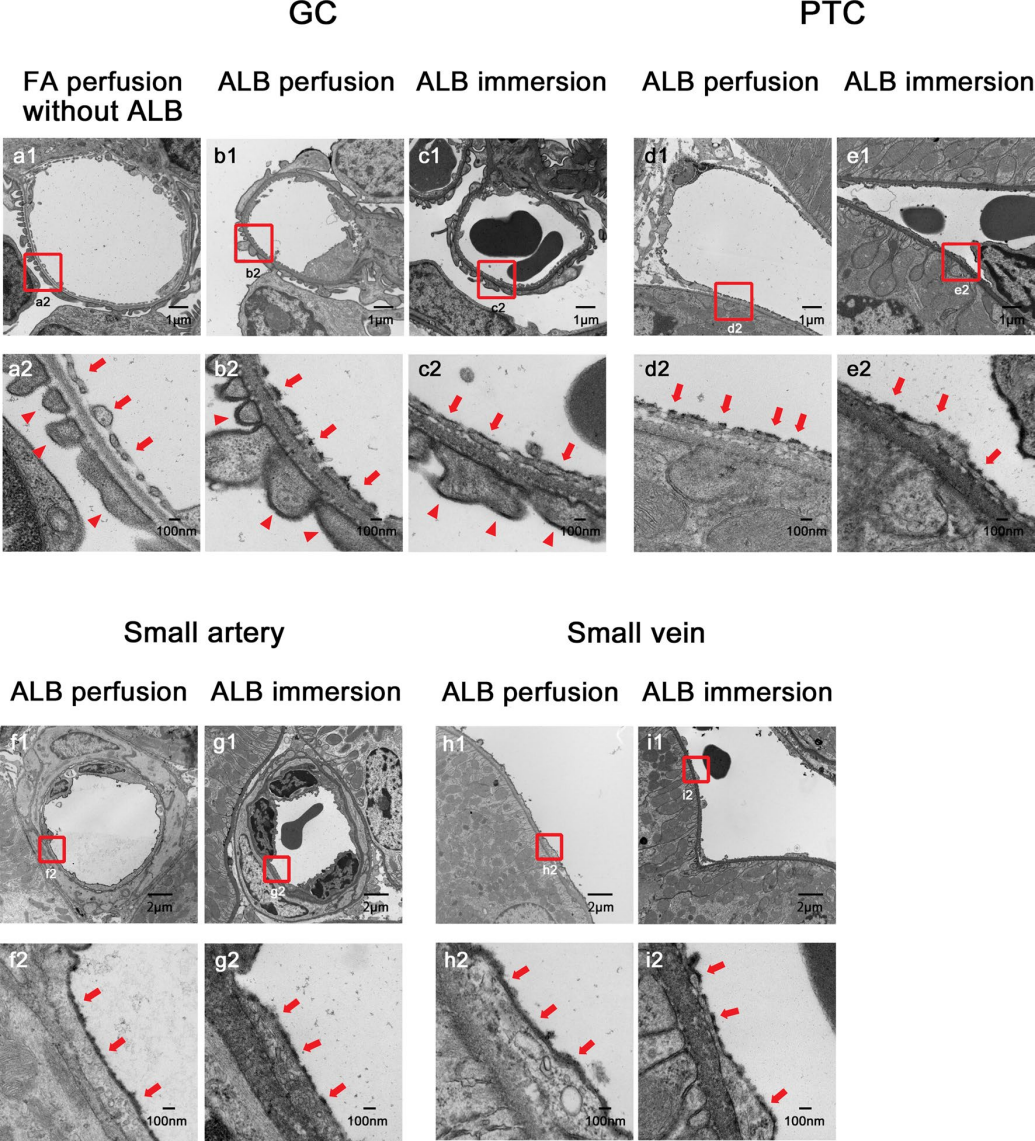
Transmission electron microscopy (TEM) images of endothelial GCX with perfusion and immersion fixation with ALB. TEM observation of the kidney specimens perfused and immersed with ALB fixative similarly demonstrated fine granular osmiophilic particles attached to the luminal surfaces. GC perfused with ALB ( −) fixation showed no particles on the surfaces of endothelial cells and podocytes (**a1**, **a2**). By contrast, GC perfused with ALB ( +) fixative exhibited a fine granular electron-dense layer on the surface of endothelial fenestrations and podocyte foot processes (**b1**, **b2**). Similar electron-dense layers were observed in GC with immersion fixation (**c1**, **c2**). Furthermore, similar fine granular electron-dense layers were also observed in the PTC with perfusion (**d1**, **d2**) and immersion (**e1**, **e2**), in the SA with perfusion (**f1**, **f2**) and immersion (**g1**, **g2**), and in the SV with perfusion (**h1**, **h2**) and immersion (**i1**, **i2**). A careful observation demonstrated that ALB particles were detached from the sites of endothelial fenestration in GC (**b2**, **c2**) and PTC (**d2**, **e2**). The particles on the podocyte surface were slightly higher in electron density and clearer in demarcation as compared with those on the endothelial surface (**b2**). Magnifications: **a1**–**e1**: 5000 × ; **a2**–**e2**: 30,000 × ; **f1**–**i1**: 3000 × ; **f2**–**i2**: 30,000 ×

TEM observation of the kidney specimens perfused with ALB fixative demonstrated fine granular osmiophilic particles attached to the luminal surfaces of GC (Fig. 6b1, b2), PTC (Fig. 6d1, d2), SA (Fig. 6f1, f2), and SV (Fig. 6h1, h2). They formed a thin layer of 30–80 nm thickness on endothelial surfaces. A careful observation using TEM demonstrated that the osmiophilic particles that correspond to GCX were detached from the sites of endothelial fenestration in GC (Fig. 6b2, c2) and PTC (Fig. 6d2, e2). Adhesion of the particles was also found on the surface of podocytes (Fig. 6b2 arrowhead). No particles were observed on the endothelial cells and podocytes in the specimens perfused with ALB ( −) fixative (Fig. 6a1, a2). There was no significant difference in the thickness of GCX between the endothelial side (39.1 ± 8.27 nm) and podocyte side (38.5 ± 6.36 nm) (*p* = 0.722), but the particles on the podocyte exhibited fine and high electron density with clear demarcation, as compared with those on the endothelial surface (Fig. 6b2).

### Endothelial GCX imaging of the kidney vasculature immersed in ALB fixative

#### LM (Fig. 7)

**Fig. 7:**
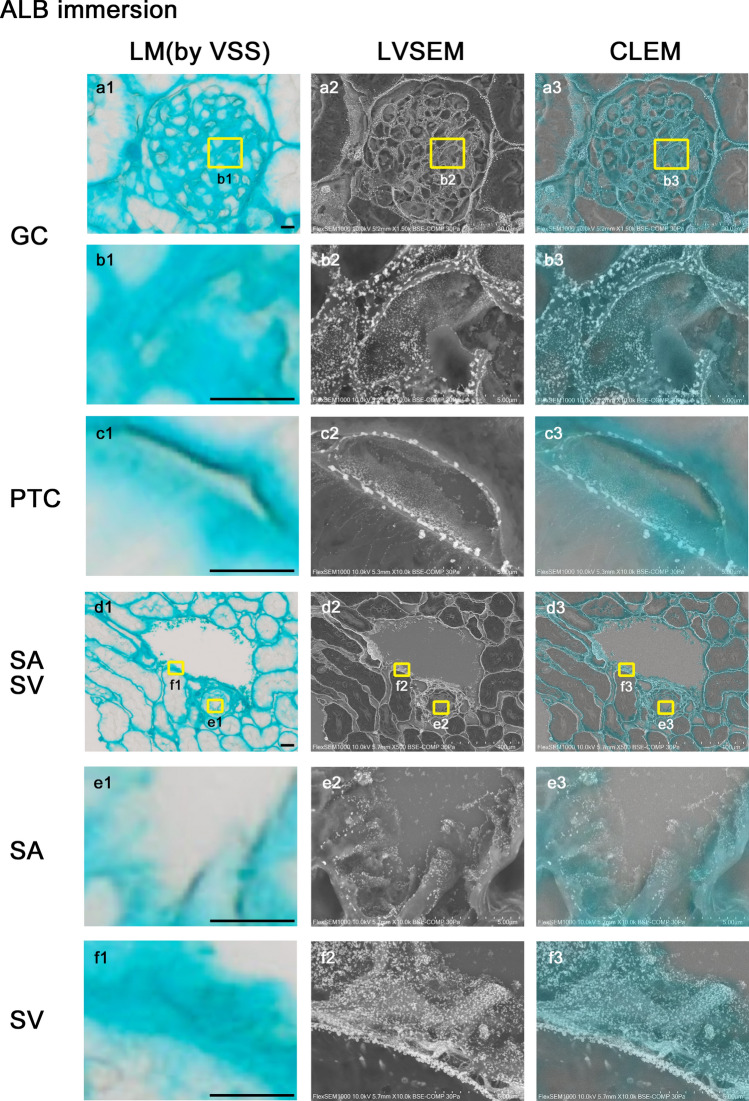
Endothelial GCX imaging of kidney vasculature immersed in ALB fixative. The kidneys immersed with ALB fixative were observed using light microscopy and a virtual slide system (**a1**–**f1**), LVSEM (**a2**–**f2**), and CLEM (**a3**–**f3**). The vessel walls of GC (**a1**, **b1**), PTC (**c1**), SA (**d1**, **e1**), and SV (**d1**, **f1**) has intense blue staining. In LVSEM, silver-enhanced particles that adhered to the vascular endothelial surfaces were observed as clearly as with perfused fixation. After merging of the LM and LVSEM images, the areas with positive of ALB staining coincided with the sites of silver-enhanced particle attachment, demonstrating the GCX layers of GC (**b3**), PTC (**c3**), SA (**e3**), and SV (**f3**) in CLEM images. Magnification; **a1**-3: 1500 × ; **b1**-3, **c1**-3: 10,000 × ; **d1**-3: 500 × ; **e1**-3, **f1**-3: 10,000 × . Scale bars: 5 μm

The kidney tissue had strong staining of ALB when using immersion fixation with ALB fixative (Fig. 7a1, d1). Although the exact staining site was not clearly determined using LM, the surface of GC, PTC, SA, SV, and Bowman’s capsule all had blue staining, and similar results were seen using perfusion fixation (Fig. 7b1, c1, e1, f1). The staining feature of the inner surface of SA was weaker than those of the other sites (Fig. 7e1).

#### LVSEM (Fig. 7)

Similar to perfusion fixation, ALB particle attachment to GCX was enhanced by silver impregnation of PAM stain and could be visualized using LVSEM (Fig. 7a2–f2). In immersion fixation, the silver-enhanced particles adhered to the endothelial surfaces, exhibited partial aggregation, and had reduced numbers of particles in GC (Fig. 7a2, b2), PTC (Fig. 7c2), and SA (Fig. 7d2, e2) as compared with those of perfusion fixation. However, in SV, the density of silver-enhanced particles was higher than in the perfusion fixation (Fig. 7f2). Adhesion of silver-enhanced particles was not seen in the specimens immersed in ALB ( −) fixative with PAM staining (data not shown).

#### CLEM (Fig. 7)

When the light microscope and LVSEM images of the samples that underwent immersion fixation were merged, the site positive for ALB staining coincided with the GCX site. Similar findings were also observed in the samples that underwent perfusion fixation (Fig. 7a3–f3).

#### TEM (Fig. 6)

TEM observation of the kidney specimens immersed with ALB fixative also showed fine granular osmiophilic particles attached to the luminal surfaces of GC (Fig. 6c1, c2), PTC (Fig. 6e1, e2), SA (Fig. 6g1, g2), and SV (Fig. 6i1, i2). This was also observed in the specimens perfused with ALB fixative. The fine granular osmiophilic particles also formed a thin layer of 30–80 nm thickness on the surfaces of endothelial cells. Particles also adhered to the surface of the podocytes (Fig. 6c2 arrowhead).

### Quantitative analysis of GCX thickness by TEM: a comparison between the samples that underwent perfusion and immersion fixation (Fig. 8)

**Fig. 8:**
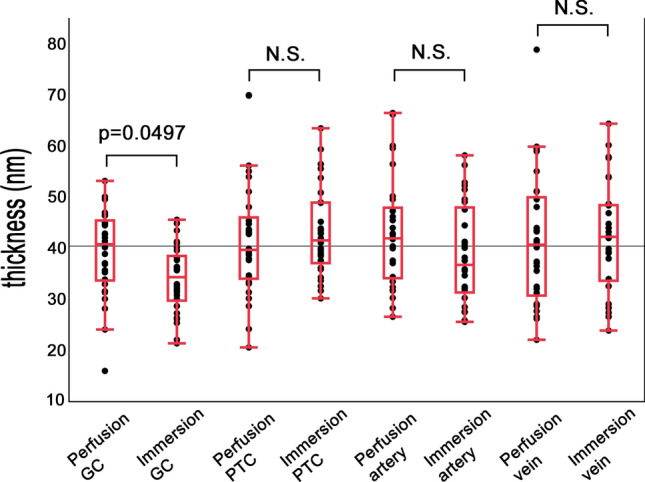
Quantitative analysis of GCX thickness by TEM. Comparisons of the GCX thicknesses of GC, PTC, SA, and SV between the specimens perfused and immersed with ALB fixative. The thickness of GCX was measured using TEM images. The average GCX thickness on GC of the perfused kidney was significantly greater than that of the immersed kidney (39.1 ± 8.27 nm in perfusion vs. 34.1 ± 6.42 nm in immersion, *p* = 0.0497). There were no significant differences in the average GCX thicknesses in PTC (41.8 ± 11.4 nm in perfusion vs. 43.0 ± 8.48 nm in immersion, *p* = 0.651), SA (43.1 ± 10.6 nm in perfusion and 38.8 ± 9.60 nm in immersion, *p* = 0.0953), and SV (41.5 ± 12.8 nm in perfusion and 42.3 ± 10.7 nm in immersion, *p* = 0.0773) between the perfused and immersed specimens. Data are presented as mean ± SD, *p* < 0.05

The thickness of ALB particles attached to the endothelial surface was considered as endothelial GCX and was morphometrically evaluated using TEM images from the samples that underwent perfusion fixation and immersion fixation at various levels of the kidney vasculature (Fig. 8). The mean GCX thickness of GC was significantly thicker with perfusion fixation than in immersion fixation (39.1 ± 8.27 nm in perfusion vs. 34.1 ± 6.42 nm in immersion, *p* = 0.0497) (Fig. 8). However, the mean thickness of PTC tended to be thicker in the immersion samples as compared with those of the perfusion samples without statistical significance (41.8 ± 11.4 nm in perfusion vs. 43.0 ± 8.48 nm in immersion, *p* = 0.651). The mean GCX thicknesses of SA and SV were not significantly different between the samples (SA: 43.1 ± 10.6 nm in perfusion and 38.8 ± 9.60 nm in immersion, *p* = 0.0953) (SV: 41.5 ± 12.8 nm in perfusion and 42.3 ± 10.7 nm in immersion, *p* = 0.0773) (Fig. 8).

## Discussion

Endothelial GCX has various biological activities, including cell adhesion, migration, differentiation, and embryogenesis. The endothelial GCX can be injured under various pathologic conditions, including sepsis, inflammation, operative stress, and diabetes [1–4]. Several clinical biomarkers for GCX injury have been reported, such as serum concentration of syndecan-1, heparin sulfate, and hyaluronic acid, reflecting the degrees of their shedding off from the endothelial surface into the bloodstream [22–24]. These markers may be useful for the evaluation of systemic injury of endothelial GCX, but evaluation of local injury in each organ and tissue is necessary for the evaluation of pathogenesis of organ-specific diseases associated with endothelial injury. Thus, morphological detection in each organ and tissue is important for the assessment of GCX injury.

As GCX is a thin layer that ranges from 50 to 1000 nm under normal conditions, EM is necessary to detect GCX. However, because of fragility of its components, a specialized EM technique is required for its detection. Based on the negatively charged properties of GCX, cationic metals or substrates, including lanthanum [5–9] and ALB [5, 6, 8, 10–12] were initially used for electron microscopic detection of GCX, with ruthenium red also being used more recently [12, 13]. As these techniques to visualize GCX are typically used for TEM, the procedures are time and labor intensive. Special equipment and techniques, such as the freeze-fracture method, are also required for SEM. Recently, LVSEM has been introduced to the field of pathological diagnosis of human kidney diseases using FFPE sections of kidney biopsy specimens [25–27]. This has allowed the observation of routine pathological specimens using SEM with a more simple and convenient technique. The advantages of this technique for EM are that it requires less time for EM specimen preparation, provides greater convenience for the use of the same sample for LM, allows a wide range of observations using a large specimen for LM, allows three-dimensional observation and it can be used on previously obtained pathological samples (FFPE blocks). In the present study, we investigated and established a method to detect endothelial GCX by LVSEM using FFPE sections fixed by perfusion and immersion of ALB-containing fixative.

To visualize GCX by TEM, previous reports recommended the use of lanthanum, osmium tetroxide, uranyl acetate, and lead citrate to obtain higher electron density [5, 6, 8, 10, 11]. We tested the use of these heavy metals to detect ALB on the endothelial surface by LVSEM, but we could not detect endothelial GCX. We next attempted to use silver enhancement to detect ALB, according to the previous reports of ultra-histochemical detection of ALB using sulfide–silver reaction [28] and of histochemical detection of glycosaminoglycans and proteoglycans by ALB staining combined with silver treatment [29–34]. Among the several silver staining methods, PAM is a major silver staining used for kidney biopsy specimens and is routinely used in many laboratories. Inaga et al. established a method to observe kidney biopsy specimens by LVSEM using FFPE sections stained with PAM [26]. This method also allows evaluation of GBM alterations by PAM. For these reasons, we used the PAM stain for the silver enhancement of ALB in this study. The silver enhancement associated with PAM staining enables the detection of ALB particles as well as the visualization of the structures and alterations of GBM. This will be useful for future studies seeking to evaluate GBM and GCX abnormalities observed in specific diseases, such as diabetic glomerulopathy or membranous nephritis.

Perfusion of fixative that contains cationic substrates has been thought to be an ideal method to assess endothelial GCX. Immersion fixation was also tested to determine whether endothelial GCX can be visualized to a similar extent as it was using perfusion fixation. Use of immersion fixation would be beneficial as it would allow the utilization of specimens obtained from human from various clinical investigations. Behnke O. et al. reported that immersion with fixative containing ALB for 1–18 h did not effectively allow visualization of endothelial GCX via EM, possibly because of the insufficient immersion of ALB into the specimens with the time and conditions used [10]. In the present study, we revealed that the pH (pH 6.0), temperature (4 °C), and duration (5 days) of fixation were necessary for efficient immersion of ALB into the specimens. The pH and temperature of ALB fixative influenced the solubility of ALB in the fixative solution. These conditions are important to prevent aggregation of ALB particles, which can easily occur without optimal conditions. The osmolality of ALB fixative and ALB (–) fixative were measured. ALB fixative had an osmolality of 1751 mOsm/L, while ALB ( −) fixative had an osmolality of 1670 mOsm/L. These osmolalities were not significantly different from that of a routine fixative, which contains 10% phosphate buffered formalin. There was no significant tissue shrinkage after the immersion periods of 1, 3, 5, and 7 days, and the effect of osmotic pressure was considered negligible.

We also evaluated the thickness of endothelial GCX by TEM and found that the thickness of endothelial GCX in various areas of the renal vasculature was around 40 nm. This is consistent with the results of previous studies that assessed the thickness of endothelial GCX using different techniques and a variety of organs. Luft JH et al. reported a thickness of 20 nm in the diaphragm capillary in mice using TEM [13], and van den Berg BM et al. reported GCX thickness as 200–500 nm in the capillary of cardiac muscle in mice using TEM [6]. Yang X. et al. found that GCX thickness in the capillaries of the epididymis muscle and mesentery was 800 nm using intravital microscopy [35]. These differences may derive from the fragile properties of GCX and its degradation that may easily occur depending on the fixation and detection conditions used in sample processing.

To evaluate the efficacy of GCX detection using two fixation methods, we compared the thicknesses of endothelial GCX at different levels of the kidney vasculature in perfused and samples fixed by immersion. In GC, GCX thickness in perfusion samples was larger than that in the immersion samples. This is possibly due to the easy binding of ALB to the vascular lumens during perfusion, in which the glomeruli are located upstream of delivery. In contrast, ALB contains a fixative, which made it difficult to access the inner portions of the glomerular structure. Accessing structures via ALB may not significantly differ between perfusion and immersion if applied to other vessels than glomerular capillary. The thickness of GCX obtained from perfusion fixation appears to reflect its correct physiological state when compared with immersion fixation method, because additional time is needed for ALB fixative to penetrate into GC during immersion. This may reduce the negatively charged property of GCX and the attached number of ALB particles on the endothelial surface. Further investigation is important to assess the differences in GCX detection using these two different fixation techniques.

Podocytes are also coated by a thin layer of negatively charged glycoproteins that are also considered GCX [16, 36, 37]. One of the major molecules in the podocyte GCX is podocalyxin, a membrane-bound glycoprotein that plays a role in the glomerular filtration charge barrier that is associated with adhesion to GBM, maintenance of slit membranes, and interaction with integrin. Podocalyxin also regulates podocyte adhesion, migration, and differentiation in normal and many pathological conditions [38]. Okada H. et al. observed the podocyte GCX via SEM and TEM and described its similarity to endothelial GCX [9]. In the present study, we also observed a thin layer of ALB particles on the surface of podocytes and endothelial cells using both perfusion and immersion fixation and LVSEM and TEM. Our observation by TEM revealed that GCX was of a similar thickness on both cells, but had a higher electron density with clear demarcation in podocyte GCX as compared with endothelial GCX. Furthermore, by LVSEM, we observed coarse and irregular aggregation of ALB granules attached to the podocytes as compared with those attached to the endothelial cells. These differences between podocyte and endothelial GCX may reflect the different composition of GCX on the different cell types. Further investigation is required to clarify the detailed three-dimensional appearances of GCX on various cells and under various conditions.

Evaluation of endothelial GCX is useful for understanding the pathogenesis of human diseases associated with endothelial injury and vascular hyperpermeability. The structure of endothelial GCX is injured in sepsis [9, 22–24, 35], and its thickness is reduced in experimental animal diabetic models [39–44]. In these studies, the specimens were perfused with a cationic substrate to detect endothelial GCX, which limits its use in animal experiments. Recently, Tachi M. et al. reported a trial to evaluate GCX in human colorectal cancer tissue and showed GCX surrounding cancer cells and the endothelial GCX of cancer vessels using immersion fixation with lanthanum [45]. Detection of GCX by immersion fixation is important to enhance the study of endothelial GCX in various human diseases. Of course the perfusion method is superior to the immersion method to detect endothelial GCX; however, we believe that the immersion method can also capture the approximate state of GCX. In the present study, we established a simple method to detect endothelial GCX and observe its three-dimensional structure by LVSEM, which can be applied to various human pathological samples. Furthermore, the CLEM method provided us with visual and comprehensive images of endothelial GCX. Further investigation is needed to overcome these limitations and to evaluate the features of endothelial GCX, including the density, distribution, and number of particles in various organs and tissues. This information will provide insights into the pathogenesis of endothelial injury and vascular hyperpermeability, in diseases such as diabetic nephropathy, nephrotic kidney diseases, infection, autoimmune and toxic diseases, and cancer, in various organs and clinical situations.

This study has several limitations. First, we could not directly evaluate GCX itself. We indirectly evaluated it by visualizing ALB particles attached to the negatively charged GCX. Alterations in GCX must be evaluated in terms of various properties, including the molecular and physiological characteristics of composed biomolecules. Second, there were morphological limitations associated with the use of FFPE specimens, which were derived from formaldehyde fixation and immersion in alcohol and xylene prior to heating for paraffin embedding. These conditions were associated with discrepancies in ultrastructural preservation. Third, we could not evaluate the morphology of the cellular components through LVSEM, because the cell membrane cannot be visualized because of silver impregnation from PAM staining. For the evaluation of cellular alterations via LVSEM, additional staining with heavy metals, such as osmium or platinum, is required. Fourth, perfusion fixation can only be used on animal specimens, but not on human biopsy specimens, which limits the physiological and exact evaluation of fragile GCX. Finally, we were only able to study normal animal tissues and could not compare our morphological findings to human tissues and some diseases. Further studies are required using samples from animal disease models and specimens obtained from diseased human tissues.

## Conclusions

We established a simple method for three-dimensional visualization of endothelial GCX using LVSEM on FFPE sections. The specimens were immersed with ALB-containing fixative and stained with PAM for visualization of ALB under LVSEM. This simple method can be applied to various human pathological samples and allows the study of the role of GCX in various diseases that are associated with endothelial injury, such as diabetes, inflammation, and cancer in future.
